# A phase 1 dose escalation and expansion study of Tarextumab (OMP-59R5) in patients with solid tumors

**DOI:** 10.1007/s10637-018-0714-6

**Published:** 2018-12-28

**Authors:** David C. Smith, Rashmi Chugh, Amita Patnaik, Kyriakos P. Papadopoulos, Min Wang, Ann M. Kapoun, Lu Xu, Jakob Dupont, Robert J. Stagg, Anthony Tolcher

**Affiliations:** 1 University of Michigan Comprehensive Cancer Center, Ann Arbor, MI USA; 2 University of Michigan Health System, 7302 Cancer Center, SPC 5946, 1500 East Medical Center Drive, Ann Arbor, MI 48109 USA; 3 START, San Antonio, TX USA; 4 OncoMed Pharmaceuticals, Inc., Redwood City, California USA

**Keywords:** Phase 1, Tarextumab, Notch inhibition

## Abstract

*Purpose* This Phase I trial evaluated the maximum tolerated dose, safety, pharmacokinetics, pharmacodynamics and preliminary efficacy of tarextumab (OMP-5948), a novel cross-reactive antibody which binds and selectively inhibits signaling via both Notch2 and Notch3, in adult patients with advanced malignancies. *Methods* Standard 3 + 3 design with tarextumab 0.5, 1, 2.5, or 5 mg/kg weekly, or 5, 7.5, or 10 mg/kg every other week, or 7.5 mg every 3 weeks. Dose-limiting toxicities (DLT) were assessed during the first 28 days. *Results* Forty-two patients received tarextumab (21 weekly, 15 every other week, 6 every three weeks). 2/6 subjects at the 5 mg/kg weekly dose, 2/3 at 10 mg/kg every other week, and 0/6 at 7.5 mg/kg every three weeks had a DLT. The maximum tolerated dose (MTD) was 2.5 mg/kg weekly, and 7.5 mg/kg on the every other and every three week schedules. Gastrointestinal (GI) toxicity was the most common adverse event with diarrhea (81%), fatigue (48%), nausea (45%), anorexia (38%), and vomiting (38%) and abdominal pain and constipation (24% each). Biomarker analysis showed regulation of stem cell and Notch gene signaling. *Conclusion* Tarextumab was generally well-tolerated at doses <2.5 mg weekly and 7.5 mg/kg every other and every third week. Diarrhea was dose-limiting above these levels, but relatively easily managed at lower doses. Inhibition of Notch pathway signaling was demonstrated at these doses. ClinicalTrials.gov Identifier: NCT01277146.

**Electronic supplementary material:**

The online version of this article (10.1007/s10637-018-0714-6) contains supplementary material, which is available to authorized users.

## Introduction

The Notch signaling pathway plays a critical role in determining cell fate, survival, and self-renewal in both normal and neoplastic tissues. Alterations in Notch signaling result in dysregulation of cellular functions including proliferation, differentiation, oncogenesis, and the maintenance of cells within tumors with stem-cell properties, specifically, the ability to self-renew, differentiate into multiple cell types, and relative chemo- and radio- resistance [1]. Composed of four receptors (Notch 1-4) and 5 ligands (Jagged 1, 2 and Delta-like ligands (DLL) 1, 3 and 4), the diversity of receptors and ligands allows an array of tissue specific effects [2]. Key among these are Notch2 and Notch3 where altered signaling via these receptors has been associated with multiple human tumors including lung, breast, ovarian, pancreatic and colon cancers.

Tarextumab (OMP-59R5) is a novel cross-reactive antibody which binds and selectively inhibits signaling via both Notch2 and Notch3 [3]. When given alone and in combination with chemotherapy, tarextumab markedly inhibited tumor growth in minimally passaged human xenograft models of pancreatic, breast, ovarian and small- cell lung cancer by inhibiting tumor proliferation and promoting differentiation. Tarextumab also inhibited tumor regrowth, the number of cells expressing stem-cell markers, and the number of tumor-initiating cells on limiting dilution assays when compared to chemotherapy alone. Gene expression assays confirmed down-regulation of the Notch pathway as well as downstream target genes involved in the epithelial-to mesenchymal transition and cell proliferation.

Based on these preclinical data, we conducted a multicenter, open-label, dose- escalation phase 1 trial of this first-in-class anti-Notch2/3 antibody, tarextumab. The study objectives were to determine the safety, pharmacokinetics, immunogenicity, and preliminary efficacy of tarextumab in patients with advanced malignancies, and to explore biomarkers of drug effect.

## Patients and methods

The study was registered with ClinicalTrials.gov (NCT01277146) and the original and amended study protocol and informed consent documents were reviewed and approved by the Institutional Review Boards of the participating institutions. Written informed consent was obtained from all patients prior to participation in any study related activities.

### Patients

Eligible subjects were at least 18 years of age and had a histologically confirmed metastatic or unresectable malignancy, an Eastern Cooperative Oncology Group (ECOG) performance status < 2, life expectancy of more than 3 months, adequate bone marrow (absolute neutrophil count (ANC) >1000/mL, hemoglobin (Hgb) >9.0 g/dL, and platelets >100,000/mL), hepatic (total bilirubin <1.5 X institutional upper limit of normal (ULN), AST (SGOT) and ALT (SGPT) < 3 X ULN (< 5 x ULN in the case of hepatic metastases)), prothrombin time (PT) and partial thromboplastin time (PTT) (≤ 1.5 X ULN), and renal function (creatinine ≤1.5 X institutional ULN or creatinine clearance 60 mL/min/1.73 m^2^ for subjects with creatinine levels above ULN). In addition, subjects were required to have measurable disease. Potential subjects who had received their last chemotherapy, biologic, or investigational therapy less than 4 weeks prior to enrollment (6 weeks if the last regimen included carmustine [BCNU] or mitomycin C), a history of allergic reactions to monoclonal antibody therapy, brain metastases, significant gastrointestinal (GI) disease including but not limited to, inflammatory bowel disease or unresected tumors involving the GI lumen, known HIV infection, bleeding disorder or coagulopathy, anticoagulation, uncontrolled hypertension or taking more than 2 antihypertensives, or with evidence of ischemia or > grade 2 ventricular arrhythmia on electrocardiogram were excluded. Pregnant or nursing women were also excluded, and women of childbearing potential were required to have had a prior hysterectomy or have a negative serum pregnancy test and be using adequate contraception prior to study entry and have agreed to use adequate contraception from study entry through at least 6 months after discontinuation of study drug. Men were required to agree to the same requirements for contraception.

### Study design

The study was designed as a standard 3+3 dose escalation trial. DLT was defined as any > grade 3 adverse event that occurred during the first 28 days, as assessed by the

National Cancer Institute Common Toxicity Criteria for Adverse Events (NCI CTCAE) version 4.02, unless it could be clearly attributed to another cause. The MTD was defined as the highest dose at which 0–1 of 6 patients experienced a DLT. All patients in a cohort were required to complete their day 28 assessment before dose escalation. Doses were initially 0.5, 1, 2.5, and 5 mg/kg weekly for 9 doses. The protocol was subsequently amended with a modification of the DLT definition to exclude grade 3 infusion reactions resolving within 24 hours, grade 3 diarrhea, nausea, and/or vomiting responding to standard medical treatment within 48 hours, and grade 3 electrolyte disturbance responding to correction within 24 hours. As the study progressed and toxicity and pharmacokinetic data became available, the protocol was amended to include a bowel management program consisting of loperamide administered at the time of the first loose stool and 2 mg with every subsequent unformed stool up to a maximum of 16 mg per day was added. Subsequently, additional cohorts at 5, 10, and 15 mg/kg IV every other week with the potential for enrolling intermediate dose levels and alternate schedules of every 2 or 3-week dosing were also added to the study.

### Study assessments

Safety was monitored with weekly physical examination, vital signs, clinical laboratory testing (complete blood counts and comprehensive metabolic panel), and assessment of performance status. Electrocardiogram (ECG), blood draw for anti-tarextumab antibody levels and urinalysis were performed every 28 days. CT or MRI was performed at baseline and every 8 weeks. Response was assessed by RECIST 1.1 [4]. Adverse events were monitored continuously from the time of enrollment through 30 days after the last dose of tarextumab or until resolution of treatment-related events which ever was longer. These events were graded using the NCI CTCAE version 4.02.

### Pharmacokinetics

Pharmacokinetic specimens were obtained at baseline, end of infusion, 0.5, 1, 3, 6, 24, 72, and 168 hours post-infusion on Study Days 0 and 49 on the weekly schedule and at baseline, end of infusion, 0.5, 1, 3, 6, 24, 72, and 168 hours post-infusion on Study Days 0 and 42 of the every other week and every 3 week schedules. Pre- and post- infusion samples were drawn on all other infusion days and a sample was obtained on any visit not associated with an infusion and at treatment termination. Plasma was harvested with sodium heparin as anti-coagulant, and analyzed for tarextumab concentration in a fully validated antigen binding enzyme-linked immunosorbent assay

(ELISA). Noncompartmental analysis (NCA) was conducted on samples from individual subjects with evaluable PK data. The PK parameters reported were Cmax, Tmax (time of maximum concentration), AUClast (area under the concentration-time curve from the first to the last observation), AUCinf (area under the concentration-time curve from the first observation to the extrapolated time infinity), AUC % Extrap (percentage of AUCinf that was extrapolated), Vss, CL, T1/2.

### Immunogenicity

Serum samples were obtained for immunogenicity assessments, at baseline, every 4 weeks while the subject was receiving study drug, at treatment termination and then weekly for the first 4 weeks following discontinuation of study drug and then at Weeks 8 and 12 following discontinuation of study drug. Samples were analyzed for anti-drug antibody (ADA) formation with a fully validated and standard bridging format ELISA. Samples tested positive for ADA were further tested for neutralizing antibody (NAb) in a fully validated standard binding format ELISA.

### Biomarkers

Specimens (hair follicles, whole blood, and tumor biopsies) for pharmacodynamic (PD) biomarkers were obtained pretreatment at baseline and at various time points thereafter. Whole blood was collected in PAXgene tubes (BD Biosciences). Hair follicles were preserved in RNAlater (Qiagen) until extraction of RNA (PicoPure RNA Isolation Kit from LifeTechnologies). Tumor biopsy RNAs were isolated using the RNeasy

Fibrous Tissue Mini Kit (Qiagen, Valencia, CA) with DNAseI treatment as described in the manufacturer’s protocol.

Affymetrix human gene chip U133 Plus 2.0 arrays were used for profiling the gene expression levels in whole blood, hair follicles and tumor biopsies (Almac Diagnostics, CLIA certified laboratory). To obtain the expression levels of each probe set, the raw CEL files in each dataset were processed for background adjustment and signal intensity normalization with GCRobust Multi-array Average (GCRMA) algorithm in the open-source bioconductor software (www.bioconductor.org). Paired sample Linear

Model for Microarray Analysis [5] and bootstrapping were used to identify differentially regulated genes by Tarextumab treatment. In the bootstrapping analysis, the 95% CI (bias-corrected adjusted, BCa) was calculated according to standard methods, applying a nonparametric bootstrap procedure [6, 7]. Each patient was compared with his or her own pretreatment sample in a paired-sample analysis. Only those genes with an absolute fold change of greater than 1.5 and within a 95% CI were considered significant. The limits of the CIs cannot cross zero for statistical significance. Thus, for the upregulated genes, the lower confidence limit (lb) had to be greater than 1.1; for the downregulated genes, the upper confidence limit (ub) had to be less than - 1.1.

### Statistical considerations

This was a Phase 1 dose-escalation study with a standard 3+3 dose escalation. Therefore, the sample size was not statistically determined. If 1 of 3 subjects experienced a DLT, that dose level was expanded to 6 subjects. If 2 or more of the 6 subjects experienced a DLT, no further subjects were dosed at that level and 3 additional subjects were added to the preceding dose cohort unless 6 subjects had already been treated at that dose level. Subjects were assessed for DLTs from the time of the first dose through day 28. Dose escalation, if appropriate, occurred after all subjects in a cohort had completed their Day 28 DLT assessment.

All analyses were conducted using SAS Version 9.1 or higher. The general analytical approach for all endpoints was descriptive in nature. No statistical hypotheses were tested. Demographic and analytical data were summarized using traditional descriptive statistical methods. Continuous variables were summarized using the number of subjects, mean, standard deviation, median, minimum, and maximum. Categorical variables were summarized using frequency counts and percentages with percentages rounded to one decimal place. Time-to-event variables were estimated by the Kaplan- Meier method.

## Results

### Patients and treatment

A total of 42 subjects were enrolled and all subjects received at least two doses of tarextumab. Baseline characteristics of the enrolled subjects are summarized in Table 1. The age and gender distributions and number and type of prior therapies were typical of phase I trials with gastrointestinal malignancies (particularly colon cancer) being the most common disease subtype enrolled.

**Table 1:** Patient characteristics

Number enrolled	42
Age:	
Median	59.5
Range	28–90
Gender:	
Female	24
Male	18
Race:	
White	39
Black	2
American Indian/Alaska Native	1
Ethnicity:	
Not Hispanic or Latino	37
Hispanic or Latino	5
Prior therapy:	
Surgery	27
Radiotherapy	18
Chemotherapy	38
Number of prior regimens	
Median	4
Range	0–13
Tumor type:	
Colon	9
Soft tissue sarcoma	6
Breast	4
Pancreatic	3
Prostate	3
Ovarian	2
Urothelial	2
Other	13

A total of 21 subjects were enrolled on the weekly schedule, 15 on the every other week, and 6 on the every three week schedule. Three subjects were treated on the weekly schedule at 0.5, 1, and 2.5 mg/kg without DLT. At 5 mg/kg weekly one of the first three subjects enrolled developed grade 3 hypokalemia due to grade 3 diarrhea which met the definition of DLT. An additional three subjects were then enrolled at this dose level and an additional subject developed grade 3 diarrhea. Three additional subjects were then enrolled at 2.5 mg/kg with no evidence of DLT. Based on these findings 2.5 mg/kg was declared as the MTD on the weekly schedule. The protocol was then amended to incorporate a standard bowel management program and an additional three subjects were enrolled at the 5 mg/kg weekly dose. All three developed grade 1-2 diarrhea despite treatment. Dosing on the every other week schedule was initiated at the 5 mg/kg dose level and three subjects enrolled without DLT. At 10 mg/kg two of three subjects enrolled had DLT with grade 3 diarrhea indicating that the MTD had been exceeded on this schedule. An additional three subjects were then enrolled at 5mg/kg every other week without DLT. Subsequently, 6 subjects were treated with the intermediate dose level of 7.5 mg/kg administered as an IV infusion every 3 weeks without a DLT. Therefore, 7.5 mg/kg every 3 weeks was determined to be the MTD for the every 3 weeks dosing schedule. Subsequently, 6 subjects were treated with 7.5 mg/kg every other week and no DLT was reported. Hence, 7.5 mg/kg every other week was the MTD for the every other week dosing schedule.

### Safety and toxicity

Tarextumab was generally well tolerated. The median number of infusions per patient was 4.5 (range 2-16). Treatment emergent adverse events (TEAE) from any cause occurring in >5% of patients are summarized in Table 2. All but one patient experienced at least one TEAE while on study. Diarrhea was the most common event, occurring in 81% of patients, followed by fatigue (48%), nausea (45%) decreased appetite (38%) and vomiting (29%). Additional gastrointestinal adverse events included abdominal pain and constipation (24% each). Severity of the TEAE was clearly related to dose and these events were much more common in the cohorts at or above the MTD on each schedule. A total of 14 Grade 3 adverse events related to tarextumab occurred in 10 patients. In order of frequency, these events were diarrhea [9], anemia (2), hypokalemia secondary to diarrhea (1), fatigue (1), and elevation in ALT (1). There were no grade 4 or 5 treatment-related adverse events on this trial. One subject with pancreatic cancer died due to disease progression during the 30 day follow up period.

**Table 2:** Treatment-associated adverse events in >5% of subjects

Event	N (%)
Diarrhea	34 (81.0)
Fatigue	20 (47.6)
Nausea	19 (45.2)
Decreased appetite	16 (38.1)
Vomiting	12 (28.6)
Abdominal pain	10 (23.8)
Constipation	10 (23.8)
Anemia	9 (21.4)
Aspartate aminotransferase increased	9 (21.4)
Dizziness	9 (21.4)
Dyspnea	8 (19.0)
Back pain	7 (16.7)
Hypokalemia	7 (16.7)
Pain in extremity	7 (16.7)
Alanine aminotransferase increased	6 (14.3)
Alkaline phosphatase increased	6 (14.3)
Pruritus	6 (14.3)
Abdominal distension	5 (11.9)
Chills	5 (11.9)
Pyrexia	5 (11.9)
Weight decreased	5 (11.9)
Asthenia	4 (9.5)
Dehydration	4 (9.5)
Dysphonia	4 (9.5)
Epistaxis	4 (9.5)
Headache	4 (9.5)
Hypertension	4 (9.5)
Muscle spasms	4 (9.5)
Musculoskeletal chest pain	4 (9.5)
Musculoskeletal pain	4 (9.5)
Rash	4 (9.5)
Flatulence	3 (7.1)
Hypersensitivity	3 (7.1)
Edema peripheral	3 (7.1)
Thrombocytopenia	3 (7.1)
Tumor pain	3 (7.1)
Vision blurred	3 (7.1)

### Pharmacokinetics

**Fig. 1:**
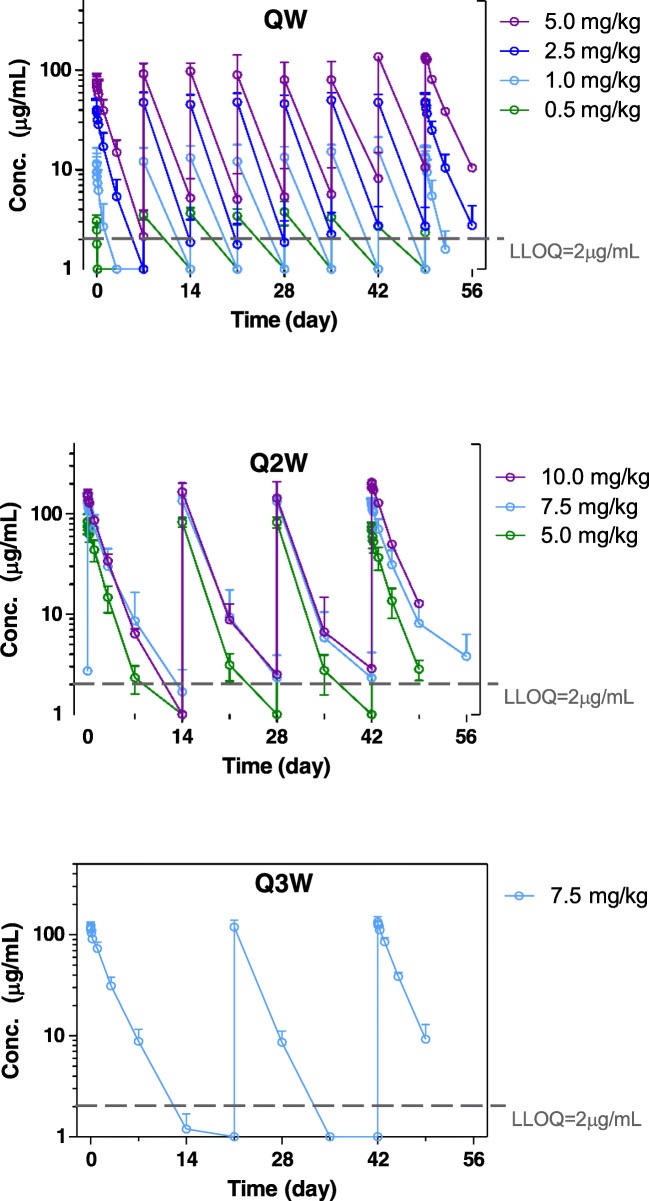
Group mean concentration-time profiles. Data organized by nominal time; sample tested below LLOQ (lower limit of quantitation) were imputed to 1 μg/mL

Pharmacokinetic endpoints were analyzed using noncompartmental analysis (NCA) on data from individual subjects with sufficient samples with measurable concentrations (N=40). Plasma concentration data from the 0.5 mg/kg weekly and 1.0 mg/kg levels is limited by low drug levels making estimation of pharmacokinetic parameters at these doses unreliable. Mean pharmacokinetic parameters are summarized in Supplemental Tables 1& 2. Minimal drug accumulation was observed in the weekly cohorts with ratios of 1.4-2.0. The ratios for the every two and three week schedules showed minimal to no accumulation (ratios 1.0-1.5). Concentration versus time curves for all three schedules are shown in Fig. 1. Clearance of tarextumab is rapid and nonlinear. Average clearance decreased from 53.8 to 25.3-28.3 mL/day/kg as the dose level increased from 2.5 mg/kg weekly to 7.5 mg/kg every two and three weeks. The dose dependent and relatively fast clearance of OMP-59R5 suggests that target mediated clearance may play an important role in the disposition of this molecule in patients at the dose level studied. The average apparent terminal half-life of tarextumab ranged from approximately 0.6 day at 1.0 mg/kg weekly to 2.0 days at 7.5 every other week and every three weeks. The trends of decreasing clearance and increasing half-life as dose increased did not appear to continue at 10 mg/kg every other week although the number of subjects is small. The steady state volume of distribution of OMP-59R5 was relatively stable across the dose levels of 2.5 and 10 mg/kg, and ranged from approximately 60 to 75 mL/kg, which suggested the molecule distributes primarily in the vascular space with modest extravasation into the tissue space. Continuous drug exposure with weekly dosing, even at relatively lower concentrations led to severe diarrhea, while intermittent dosing with a period of washout enabled dose escalation to a significantly higher dose level. However, the concentration threshold for drug washout to reduce risk for diarrhea could not be established due to limited data.

### Immunogenicity

Anti-tarextumab antibodies were detected in 26% (11/42) of subjects. Out of the 11 subjects confirmed positive for anti-tarextumab antibodies, 10 were confirmed to be positive for neutralizing antibodies in a confirmatory immunodepletion assay. Formation of these antibodies did not appear to decrease drug exposure in these subjects, although were detected around the time of treatment termination in all cases.

### Biomarkers

**Fig. 2:**
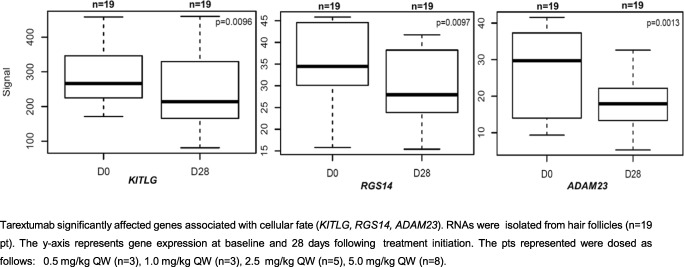
Stem cell and differentiation genes regulated in hair follicles by tarextumab treatment. Tarextumab significantly affected genes associated with cellular fate (*KITLG, RGS14, ADAM23*). RNAs were isolated from hair follicles (*n* = 19 pt). The y-axis represents gene expression at baseline and 28 days following treatment initiation. The pts. represented were dosed as follows: 0.5 mg/kg QW (*n* = 3), 1.0 mg/kg QW (n = 3), 2.5 mg/kg QW (*n* = 5), 5.0 mg/kg QW (*n* = 8)

**Fig. 3:**
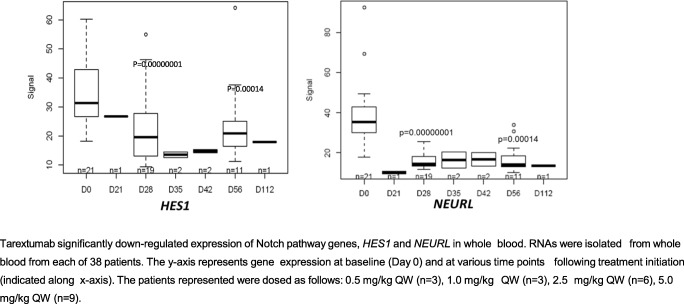
Effects of tarextumab treatment on Notch target gene expression in whole blood. Tarextumab significantly down-regulated expression of Notch pathway genes, *HES1* and *NEURL* in whole blood. RNAs were isolated from whole blood from each of 38 patients. The y-axis represents gene expression at baseline (Day 0) and at various time points following treatment initiation (indicated along x-axis). The patients represented were dosed as follows: 0.5 mg/kg QW (n = 3), 1.0 mg/kg QW (n = 3), 2.5 mg/kg QW (*n* = 6), 5.0 mg/kg QW (*n* = 9)

**Fig. 4:**
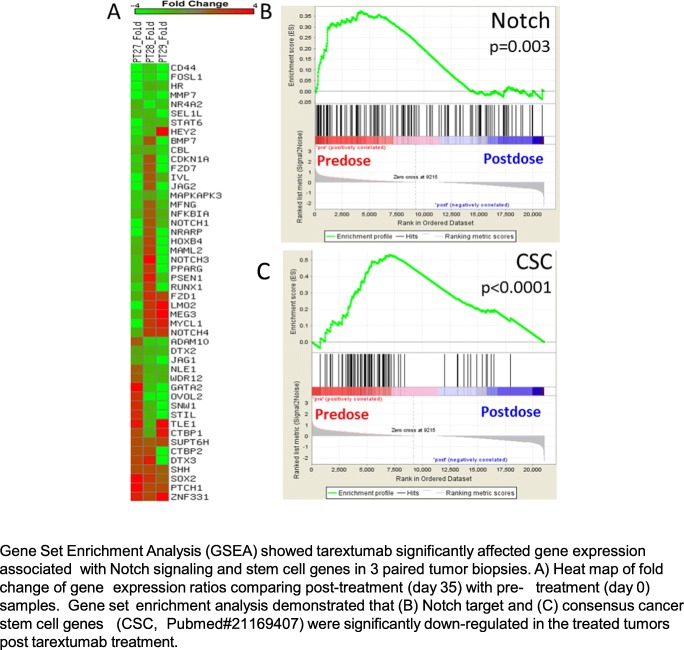
Tarextumab reduced Notch and stem cell signatures in biopsied tumors. Gene Set Enrichment Analysis (GSEA) showed tarextumab significantly affected gene expression associated with Notch signaling and stem cell genes in 3 paired tumor biopsies. **a** Heat map of fold change of gene expression ratios comparing post-treatment (day 35) with pre- treatment (day 0) samples. Gene set enrichment analysis demonstrated that (**b**) Notch target and (**c**) consensus cancer stem cell genes (CSC, Pubmed#21169407) were significantly down-regulated in the treated tumors post tarextumab treatment

Biomarker analysis of hair follicles from 19 pts showed significant regulation of stem cell and Notch genes (e.g., *KITLG*, *RGS14* and *ADAM23*) by tarextumab, consistent with cellular fate changes associated with Notch inhibition (Fig. 2). Gene expression analysis in blood from 38 pts showed a persistent decrease in Notch pathway genes *HES1* and *NEURL* post-tarextumab treatment starting at day 21 (Fig. 3). *HES1* and *NEURL* were regulated starting at doses of 1mg/kg every week and above and in all dose schedules (Supplemental Fig), with higher doses showing increased regulation of the biomarkers. Notch and stem cell signaling biomarkers as measured by gene set enrichment analysis were also decreased in three patients who underwent sequential tumor biopsies (Fig. 4).

### Efficacy

Thirty-eight subjects were evaluable for response. There were no objective responses by RECIST criteria to single agent tarextumab. Nine subjects had stable disease and six of these subjects had stable disease by RECIST lasting greater than 56 days (range: 61-165 days).

## Discussion

Tarextumab is a first-in-class anti-Notch 2/3 antibody which selectively binds these receptors. On this trial the main dose-limiting toxicity was diarrhea which correlated with the duration of drug exposure and consistent with preclinical findings. The MTD was 2.5 mg/kg on the weekly schedule and 7.5 mg/kg on both the every other and every three week schedules. Diarrhea occurred at 2.5 mg/kg weekly and higher dose levels. The incidence and severity of the diarrhea was dose dependent. Diarrhea was mostly Grade 1 or 2 at the dose levels ≤ the MTD, manageable with supportive care. 6 subjects reported Grade 3 diarrhea (n=2 at 5mg/kg weekly, n=1 at 5 mg/kg every other week, n=3 at 10 mg/kg every other week), most of which were at the dose levels above the MTD. There were no Grade 4 or 5 treatment related toxicities. The only other common toxicities experienced by subjects on this study were mild fatigue and the constellation of nausea, decreased appetite, and vomiting. The recommended phase 2 dose 7.5 mg/kg given on an every 2 or 3 week schedule.

The toxicity profile of tarextumab is similar to that seen with small molecule inhibitors of gamma secretase, the intracellular enzyme responsible for the cleavage of activated Notch receptors at the cell membrane allowing translocation of the intracellular component to the nucleus. Continuous dosing of gamma secretase inhibitors is limited by the development of secretory diarrhea presumably due to the effects of Notch pathway inhibition on progenitor cells within the intestinal crypts [8, 9]. It is also consistent with the predicted effect from preclinical studies which have shown that the fate of immature progenitor cells in the intestine is under the control of Notch signaling and that pathway blockade results in the differentiation of these cells into secretory goblet cells [10, 11]. Additional preclinical data suggest that these effects can be mitigated while preserving inhibitory effects using an intermittent schedule and or glucocorticoids [12]. Clinically this has been confirmed in trials of several agents targeting this enzyme and the pattern would appear to fit with the pharmacokinetics of the dosing schedules with longer intervals which allowed tarextumab drug washout between doses and presumably regeneration of the intestinal functions [8, 13, 14]. Biomarker analysis demonstrated evidence of Notch signaling inhibition by tarextumab in hair follicles, in whole blood, and in serial tumor biopsies.

In summary, tarextumab was well tolerated in patients with advanced solid tumors at doses of 2.5 mg/kg weekly, and 7.5 mg/kg every 14 or 21 days, with biomarker evidence of Notch pathway inhibition at these doses. Disease stabilization was seen in patients with a variety of malignancies on this trial. Phase Ib/2 studies of tarextumab in combination with chemotherapy have been conducted in patients with pancreatic cancer (NCT01647828) and small cell lung cancer (NCT01859741).

## Electronic supplementary material

ESM 1 (DOCX 136 kb)

ESM 2 (PDF 133 kb)
