# Supplementary material for: A phase 1 dose escalation and expansion study of Tarextumab (OMP-59R5) in patients with solid tumors
Source: Invest New Drugs. 2018 Dec 28;37(4):722–30. doi: 10.1007/s10637-018-0714-6 (PMC6647865; doi:10.1007/s10637-018-0714-6)
Supplement: Supplementary file 1 — (DOCX 136 kb) [file 10637_2018_714_MOESM1_ESM.docx]

**Supplemental figure:** Effects of tarextumab treatment on Notch target gene expression in whole blood by dose group


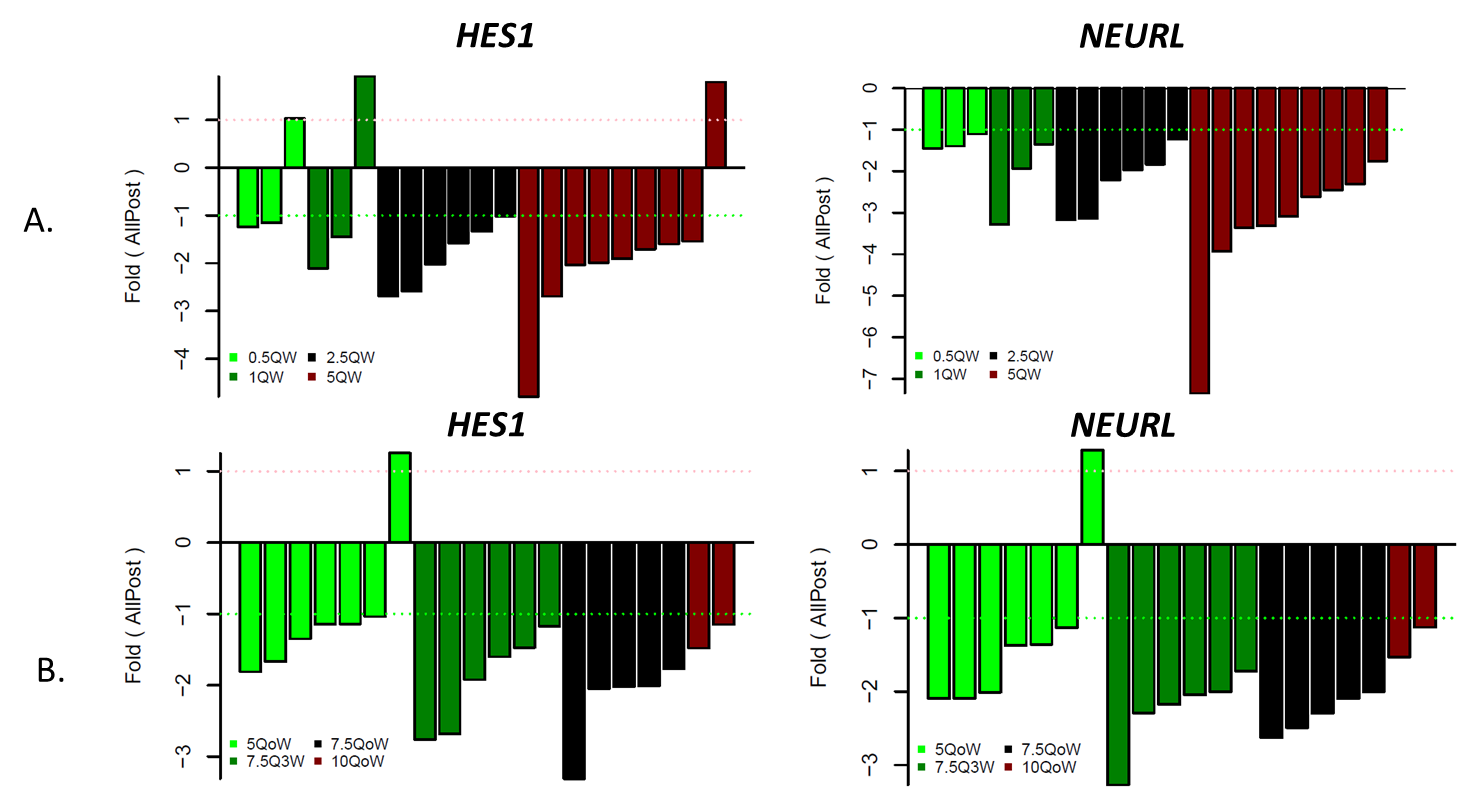


Tarextumab down-regulated expression of Notch pathway genes, *HES1* and *NEURL,* in whole blood (n=38). A) Every week dose groups (qw) and B) every other(qow) or three week (q3w) dose groups were run in separate batches and plotted separately (q3w not shown). The x-axis shows individual pts in each dose group and the y-axis shows the gene expression fold changes of all post dose samples compared with their base line samples.
