# Supplementary material for: A phase 1 dose escalation and expansion study of Tarextumab (OMP-59R5) in patients with solid tumors
Source: Invest New Drugs. 2018 Dec 28;37(4):722–30. doi: 10.1007/s10637-018-0714-6 (PMC6647865; doi:10.1007/s10637-018-0714-6)
Supplement: Supplementary file 2 — (PDF 133 kb) [file 10637_2018_714_MOESM2_ESM.pdf]

Supplemental Table 1: Mean Tarextumab Pharmacokinetic Parameters After Weekly Dosing

Dose (mg/kg)	Day	N	$T_{1/2}$ (days)	T_{max} (days)	C_{max} (µg/mL)	AUC_{last} (day*µg/mL)	AUC_{0-inf} (day*µg/mL)	AUC % Extrap (%)	CL (mL/day/kg)	V_{ss} (mL/kg)	Accumulation Ratio[c]
0.5[a]	0	1	ND	0.020	3.37	0.15	ND	ND	ND	ND	
	49	0	ND	ND	ND	ND	ND	ND	ND	ND	ND
1.0[b]	0	3	0.553	0.020	11.5	4.95	7.6	41.8	209	147.8	
	49	2	0.925	0.040	13.8	13.2	16.9	26.0	69.1	78.3	1.76
2.5	0	6	1.125	0.030	41.0	48.2	57.1	15.0	53.8	77.3	
	49	4	1.665	0.030	49.0	86.7	98.8	13.9	27.6	56.4	2.00
5.0	0	9	1.49	0.024	75.7	124.1	143.5	15.6	37.9	73.0	
	49	1	2.04	0.060	136.3	330.0	360.2	8.5	13.9	35.7	1.42

Abbreviations: AUC, area under the concentration-time curve; CL, clearance; C_{max}, maximum plasma concentration after administration; T_{max}, time to reach the observed maximum (peak) concentration; $T_{1/2}$, half-life; V_{ss}, volume of distribution at steady state.

[a] Three subjects were treated in this cohort; Subject 001001001 had serum concentration data with suspected assay interference and was not included in analysis; Subject 001001002 had all samples except 5-minute postinfusion below detection limit, thus not included in this analysis.

[b] The AUC % extrapolations were larger than 20% at this dose level.

[c] Calculated as $AUC_{last,\ Day\ 49}/AUC_{last,Day0}$.

ND: not determined due to insufficient data.

Supplemental Table 2: Mean Tarextumab Pharmacokinetic Parameters After Every Other Week and Every 3 Weeks Dosing

Dose (mg/kg)	Schedule	Day	N	$T_{1/2}$ (days)	T_{max} (days)	C_{max} (µg/mL)	AUC_{last} (day*µg/mL)	$AUC_{0\text{-}inf}$ (day*µg/mL)	AUC % Extrap(%)	CL (mL/day/kg)	V_{ss} (mL/kg)	Accumulation Ratio[a]
5.0	QoWk	0	6	1.39	0.033	87.7	**147**	154	5.17	34.2	59.1	
		42	4	1.48	0.035	75.2	127	135	6.72	42.7	74.3	1.13
7.5	QoWk	0	6	2.09	0.027	138	284	309	7.30	28.3	64.8	
		42	5	2.04	0.028	131	270	287	6.30	29.1	68.5	1.08
7.5	Q3Wk	0	6	2.03	0.030	120	285	307	6.96	25.3	64.0	
		42	3	1.74	0.070	129	317	337	5.81	22.4	50.4	1.04
10.0	QoWk	0	3	1.59	0.053	164	317	331	4.28	30.3	60.5	
		42	1	1.76	0.040	206	465	495	6.17	20.2	45.7	1.48

Abbreviations: AUC, area under the concentration-time curve; CL, clearance; C_{max}, maximum plasma concentration after administration; QoWk, every other week; Q3Wk, every 3 weeks; T_{max}, time to reach the observed maximum (peak) concentration; $T_{1/2}$, half-life; V_{ss}, volume of distribution at steady state.

[a] Calculated as $AUC_{last,\ Day\ 42}/AUC_{last,Day0}$.
